# The Snake with the Scorpion’s Sting: Novel Three-Finger Toxin Sodium Channel Activators from the Venom of the Long-Glanded Blue Coral Snake (*Calliophis bivirgatus*)

**DOI:** 10.3390/toxins8100303

**Published:** 2016-10-18

**Authors:** Daryl C. Yang, Jennifer R. Deuis, Daniel Dashevsky, James Dobson, Timothy N. W. Jackson, Andreas Brust, Bing Xie, Ivan Koludarov, Jordan Debono, Iwan Hendrikx, Wayne C. Hodgson, Peter Josh, Amanda Nouwens, Gregory J. Baillie, Timothy J. C. Bruxner, Paul F. Alewood, Kelvin Kok Peng Lim, Nathaniel Frank, Irina Vetter, Bryan G. Fry

**Affiliations:** 1 Department of Pharmacology, Biomedicine Discovery Institute, Monash University, Clayton 3168, Australia; daryl.yang@monash.edu (D.C.Y.); wayne.hodgson@monash.edu (W.C.H.); 2 Venom Evolution Lab, School of Biological Sciences, University of Queensland, St. Lucia 4072, Australia; danieldashevsky@gmail.com (D.D.); james.dobson@uqconnect.edu.au (J.D.); tnwjackson@gmail.com (T.N.W.J.); jcoludar@gmail.com (I.K.); jordan_debono@hotmail.com (J.D.); iwanhx@yahoo.com (I.H.); 3 Institute for Molecular Bioscience, University of Queensland, St. Lucia 4072, Australia; j.deuis@uq.edu.au (J.R.D.); a.brust@imb.uq.edu.au (A.B.); g.baillie@imb.uq.edu.au (G.J.B.); t.bruxner@imb.uq.edu.au (T.J.C.B.); p.alewood@imb.uq.edu.au (P.F.A.); 4 Bejing Genomics Institute-Shenzhen, Shenzhen 518083, China; xiebing@genomics.cn; 5 School of Chemistry and Molecular Biosciences, University of Queensland, St. Lucia 4072, Australia; p.josh@uq.edu.au (P.J.); a.nouwens@uq.edu.au (A.N.); 6 Lee Kong Chian Natural History Museum, National University of Singapore, 2 Conservatory Drive, Singapore 117377, Singapore; kelvinlim@nus.edu.sg; 7 Mtoxins, 1111 Washington ave, Oshkosh, WI 54901, USA; nate@mtoxins.com; 8 School of Pharmacy, University of Queensland, Woolloongabba 4102, Australia

**Keywords:** toxicofera, venom, evolution, neurotoxin, sodium channel, pharmacology

## Abstract

Millions of years of evolution have fine-tuned the ability of venom peptides to rapidly incapacitate both prey and potential predators. Toxicofera reptiles are characterized by serous-secreting mandibular or maxillary glands with heightened levels of protein expression. These glands are the core anatomical components of the toxicoferan venom system, which exists in myriad points along an evolutionary continuum. Neofunctionalisation of toxins is facilitated by positive selection at functional hotspots on the ancestral protein and venom proteins have undergone dynamic diversification in helodermatid and varanid lizards as well as advanced snakes. A spectacular point on the venom system continuum is the long-glanded blue coral snake (*Calliophis bivirgatus*), a specialist feeder that preys on fast moving, venomous snakes which have both a high likelihood of prey escape but also represent significant danger to the predator itself. The maxillary venom glands of *C. bivirgatus* extend one quarter of the snake’s body length and nestle within the rib cavity. Despite the snake’s notoriety its venom has remained largely unstudied. Here we show that the venom uniquely produces spastic paralysis, in contrast to the flaccid paralysis typically produced by neurotoxic snake venoms. The toxin responsible, which we have called calliotoxin (δ-elapitoxin-Cb1a), is a three-finger toxin (3FTx). Calliotoxin shifts the voltage-dependence of Na_V_1.4 activation to more hyperpolarised potentials, inhibits inactivation, and produces large ramp currents, consistent with its profound effects on contractile force in an isolated skeletal muscle preparation. Voltage-gated sodium channels (Na_V_) are a particularly attractive pharmacological target as they are involved in almost all physiological processes including action potential generation and conduction. Accordingly, venom peptides that interfere with Na_V_ function provide a key defensive and predatory advantage to a range of invertebrate venomous species including cone snails, scorpions, spiders, and anemones. Enhanced activation or delayed inactivation of sodium channels by toxins is associated with the extremely rapid onset of tetanic/excitatory paralysis in envenomed prey animals. A strong selection pressure exists for the evolution of such toxins where there is a high chance of prey escape. However, despite their prevalence in other venomous species, toxins causing delay of sodium channel inhibition have never previously been described in vertebrate venoms. Here we show that Na_V_ modulators, convergent with those of invertebrates, have evolved in the venom of the long-glanded coral snake. Calliotoxin represents a functionally novel class of 3FTx and a structurally novel class of Na_V_ toxins that will provide significant insights into the pharmacology and physiology of Na_V_. The toxin represents a remarkable case of functional convergence between invertebrate and vertebrate venom systems in response to similar selection pressures. These results underscore the dynamic evolution of the Toxicofera reptile system and reinforces the value of using evolution as a roadmap for biodiscovery.

## 1. Introduction

Squamate reptiles are a research area of great controversy and debate at all levels, including higher order organismal relationships [1]. Traditional frameworks based upon morphology [2,3,4,5,6] were dramatically over-turned by genetic studies which revealed that the morphological plasticity of these animals had obscured their authentic evolutionary relationships [7,8,9,10,11,12,13,14,15,16]. While the genetics are now well-resolved, a form of scientific culture-war persists, with some adherents of morphologically based taxonomy not modifying their position in the face of genetic evidence supporting the paradigm shifts [17]. Attempts at reconstructing the evolutionary history by using a combination of morphology, fossils, and genetics resulted in poorly resolved trees [18] that had significantly different frameworks from those reconstructed using only genetic evidence [7,8,9,10,11,12,13,14,15,16]. The utilisation of ambiguous morphological characters (which may be arbitrarily scored as divergent or convergent) creates errors relative to the use of non-ambiguous genetic data. Use of such skewed trees results in flawed conclusions when used to reconstruct the evolutionary history not only of the organisms but also their associated venom systems, such as in [17,19]. Another study which examined tissue pattern expressions of toxin and non-toxin homologues in an attempt to cast doubt on the shared ancestry of reptile venom systems was methodologically flawed due to expression values being calculated using replicate averages of values up to nearly 6,000-fold apart, only averaging *N* = 2 for many of the experiments, and including failed experiments with zero values in *N* = 3 calculations (Supplementary Tables S5–S9 of [20]).

Squamate reptiles are an extremely morphologically plastic clade, where dramatic variations in size and shape [21] may not be an accurate reflection of the organismal genetic relationships [22,23,24]. One particular clade (Toxicofera) evolved a morphological feature not otherwise described in extant squamates. The basic substrate for the evolution of reptile venom was the exaptation of existing salivary glands in the common ancestor of toxicoferan reptiles (Anguimorpha and Iguania lizards plus snakes), whereby the protein secreting region was increased in size and further segregated from the mucus secreting region [25]. These glands remained in an apparently incipient state in most Iguania except for vertebrate feeding lineages such as *Anolis equestris*, *Pogona* sp., and *Chlamydosaurus Kingii*. In snakes and anguimorph lizards, however, extensive diversification has occurred [19,25,26,27,28,29,30]. In particular, this key evolutionary innovation under-pinned the explosive diversification of the advanced snakes [10,11,12,13,14,15,27,30,31,32,33,34,35,36]. While initial venom proteins were pre-existing salivary proteins that were heightened in expression, such as CRiSP and kallikrein [27,37,38,39], the promiscuity venom glands share with other secretory tissues allowed for lineage-specific expression of other protein types [20,25,26,27,28,29,40,41,42,43,44,45,46,47,48] in addition to low-level reversal of expression of venom-gland-derived proteins in other tissues [40]. For many toxin types, particularly those with high disulphide-bond ratios relative to total amino acid content [37], evolutionary selection pressures resulted in neofunctionalisations that produced a myriad of derived activities [41].

As with many other modes of evolution, periodically the venom proteins undergo punctuated molecular evolution, dynamically diversifying into new functional forms early in their evolution, with periods of stasis after that interrupted by further periods of Gouldian punctuated evolution [49,50,51,52,53]. Examples include elapids first arriving in Australia when it was a continent largely devoid of snakes other than slow moving pythons and burrowing blind snakes [54]. Subsequent explosive organismal diversification was paralleled by dramatic shifts in venom chemistry even between populations with minimal organismal genetic variation [55,56].

Venom is a key evolutionary innovation, in myriad lineages, that diversifies in concert with organismal morphological diversification [11,13,14,15,25,26,27,28,29,30,33,34,37,41,43,44,45,46,47,55,56,57,58,59,60,61,62,63]. This mechanical and chemical interrelationship continues throughout the evolutionary history of a venomous lineage. In some cases, explosive morphological changes apparently occur in an evolutionarily short period of time, with the venom changing along with it in response to emergent selection pressures. Considerations of the venom delivery system must also include the morphological features associated with prey contact. Ambush feeding snakes such as most viperids rely on camouflage, which itself may be both morphological (pattern) and chemical (control of smell emission) [64]. They typically have muscular builds that facilitate fast and powerful strikes capable of driving their large, flexible fangs deep into a prey item and delivering copious amounts of venom from their bulky venom glands. Elapid snakes, however, are generally more agile as they are usually active foragers that deliver smaller amounts of very toxic venom through short, rigid fangs connected to the relatively small venom glands.

As proteinaceous toxins are large in size, they necessitate delivery via a wound, however small [26,27,58]. Thus, in addition to variation in the types and activities of proteins secreted by the venom glands, there has been extensive evolutionary tinkering of all elements of this integrated weapons system, including the glands themselves and the associated dentition. The morphological plasticity of the glands has resulted in diverse forms. Plesiomorphic anguimorph lizard glands are relatively unstructured despite containing large lumens in the well-defined protein-secreting region, but two independent lineages of anguimorph lizards have evolved completely segregated, membrane-encapsulated protein- and mucus-secreting regions [25,27,29]. In snakes there is considerable variation in oral gland form and function, while in the advanced snakes the maxillary venom glands have been fully segregated into distinct protein- and mucus-secreting tissues, accompanied by extensive variation of features such as the relative presence, size, and shape of liquid venom storage lumens [27,44,65].

While a venom delivering wound can be created by any form of tooth capable of penetrating skin, in the advanced snakes enlarged rear teeth have independently evolved on multiple occasions and also display extensive variation [44], in some cases accompanied by grooving that is convergent with that seen in other extant and extinct venomous lineages including archosauriforms [66], conodonts [67], sphenodons [68], insectivorous mammals such as shrews and solenodons [69,70,71,72], and bird-like dinosaurs hypothesised to specialise in feeding upon on early birds [73].

Refinements of the ancestral snake venom system have included, on three independent occasions, the evolution of high-pressure delivery systems terminating in hollow teeth, with the teeth in each case being derived from enlarged rear teeth from within three different non-front-fanged lineages [27,74]. Within each of these front-fanged lineages, on at least one occasion (twice within the elapid snakes) venom glands have become elongated to extend down into the neck region: *Atractaspis* within the Lamprophiidae; *Calliophis* and *Toxicocalamus* within the Elapidae; and *Causus* within the Viperidae [27]. While *Atractaspis* and *Causus* venom glands are elongations of the venom glands extending directly down the neck, *Calliophis* elongated glands differ sharply in that a long duct extends down the neck and then inside the ribs, with the glands located now inside the body cavity.

The influence of these elongations upon venom composition within each lineage has remained uninvestigated. For example, the sarafotoxin from *Atractaspis* venoms are an example of a lineage specific recruitment of protein type for use as a venom component, but it is unknown whether this precedes or is subsequent to the diversification of the venom glands into an apomorphic long-glanded state found only in one clade of *Atractaspis*, but not all. Similarly, Asian elapid snakes in the *Calliophis* genus have plesiomorphic short-glanded (*C. gracilis*) and apomorphic long-glanded forms (*C. bivirgatus* and *C. intestinalis*).

Coral snakes in the genus *Calliophis* feed upon other snakes, including other snake-eating venomous species of Elapidae such as kraits (*Bungarus*) and king cobras (*Ophiophagus*) [75,76]. A unique evolutionary scenario ensues, a chemical arms race between predator and prey in which the risk of role reversal becomes a key selection pressure driving the evolution of toxins that rapidly render prey incapable of retaliation or escape. Snakes that hunt animals capable of inflicting serious retaliatory wounds often release their intended prey after envenomation. In this situation, selection may favour the evolution of toxins that rapidly disable prey, either to prevent it moving too far to be recovered or to prevent the possibility of it attacking and injuring the snake.

**Figure 1:**
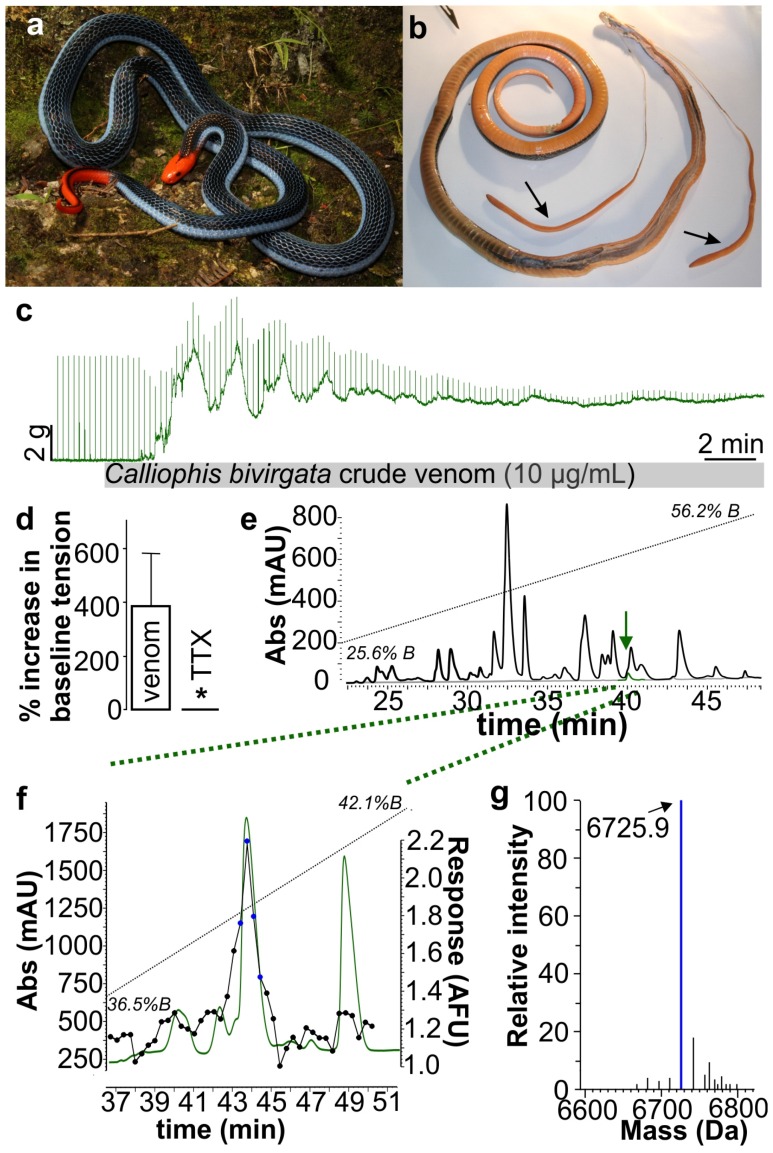
Isolation of calliotoxin (δ-elapitoxin-Cb1a), the first Na_V_ activator from snake venom. (**a**) Specimen of *Calliophis bivirgatus*, the blue coral snake (photo by Tom Charlton); (**b**) Dissected preserved 112 cm *Calliophis bivirgatus* specimen with 29 cm elongated venom glands (arrows); (**c**) *C. bivirgatus* crude venom (10 μg/mL) elicits rising contractions of the skeletal muscle in the chick biventer cervicis nerve-muscle preparation while abolishing nerve mediated contractions; (**d**) Pre-treatment with tetrodotoxin (0.1 μM) prevents the increase in baseline tension elicited by crude *C. bivirgatus* venom. * *p* Value = 0.0272, significantly different from venom alone, unpaired *t*-test; (**e**) RP-HPLC fractionation of crude *C. bivirgatus* venom (150 μg) on a BDS Hypersil C18 column. Dotted line; gradient (25.6%–56.2% solvent B). Solid grey line shows elution of purified calliotoxin (green peak) under identical conditions. Arrow indicates active fraction; (**f**) Left axis (green line): orthogonal purification of RP-HPLC fraction containing calliotoxin on a Synergi-Hydro RP column. Dotted line; gradient (36.5%–42.1% solvent B). Right axis (black line/filled circles): response of SH-SY5Y cells to corresponding 20 s-fractions. Fractions indicated by blue circles were collected for sequencing and pharmacological analysis; (**g**) The fraction containing purified native calliotoxin is dominated by a single isotopic mass of 6725.9 Da.

With its combination of electric blue dorsolateral stripes and neon red head, tail, and ventral scales, the blue coral snake, *Calliophis bivirgatus*, is arguably one of the world’s most striking species of snake (Figure 1a). An encounter with one is high on the list for many reptile enthusiasts and nature photographers visiting southern Thailand, Malaysia, Singapore, and western Indonesia. The species is of additional interest to anatomists and toxinologists studying the evolution and diversification of the snake venom system as it (along with its congener *C. intestinalis*) possesses novel elongated venom glands that extend for up to one quarter of the length of its body [77] (Figure 1b). It is also of medical significance as, in spite of only a small handful of confirmed bites, it has been responsible for at least one human fatality [76], is suspected of causing at least one more [78], and has no known antivenom. In spite of these high levels of interest, the venom has been subject to relatively few studies [79,80,81]. Those studies that examined the toxin content of the venom concluded that all the three-finger toxins present were exclusively cytotoxic in their effects [80,81]. However, this limited scope of activity attributed to the venom was reflective of the very narrow scope of assays performed and cytotoxicity was largely assumed based on similarity of partial sequences to other toxin types from other snakes rather than full activity characterisation. One study, which examined the usefulness of Taiwan antivenom, preincubated the venom with antivenom (a clinically unrealistic situation) and even then required very high doses to exert any meaningful level of inhibition [81].

## 2. Results and Discussion

**Figure 2:**
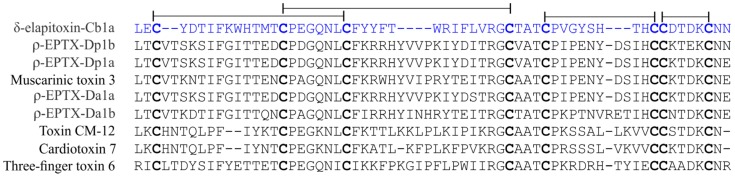
Sequence of calliotoxin (δ-elapitoxin-Cb1a) and the most closely related three-finger toxins. Homology to known three-finger toxins (3FTxs) was determined using BLAST and multiple sequence alignment conducted with CLUSTAL Omega (1.2.2). Calliotoxin (δ-elapitoxin-Cb1a; blue sequence) is most closely related to ρ-EPTX-Dp1b (P25518.1; *Dendroaspis polylepis polylepis*; 50% sequence identity), ρ-EPTX-Dp1a (P80495.1; *Dendroaspis polylepis polylepis*; 50% sequence identity), ρ-EPTX-Da1a (P85092.1; *Dendroaspis angusticeps*; 49% sequence identity), and ρ-EPTX-Da1b (P86419.1; *Dendroaspis angusticeps*; 53% sequence identity), three-finger toxins with activity at α1-adrenoreceptors, as well as muscarinic toxin 3 (P81031.2; *Dendroaspis angusticeps*; 49% sequence identity) targeting the mAChR and Toxin CM-12 (P62394.1; *Naja haje haje*; 42% sequence identity), Cardiotoxin 7 (P49122.1; *Naja atra*; 42% sequence identity), and Three-finger toxin 6 (JAS05190.1; *Micrurus tener*; 47% sequence identity) with unknown pharmacological activity. Conserved ancestral cysteines shown in bold, disulphide-bond pattern shown in black lines.

In the present study, the pharmacology of blue coral snake venom was investigated. A form of neurotoxicity, previously known from cone snail and scorpion venoms, was identified for the first time from the venom of a snake. In the indirectly stimulated chick biventer cervicis nerve-muscle assay, which is a skeletal muscle preparation, *C. bivirgatus* venom (10 μg/mL) produced large muscle contractions and fasciculations (Figure 1c) which were significantly inhibited by the addition of the sodium channel antagonist tetrodotoxin (Figure 1d; TTX; 0.1 μM) (Figure 1c). Activity-guided fractionation using SH-SY5Y human neuroblastoma cells identified a peak dominated by a single isotopic mass of 6725.89 Da as the active component (Figure 1d–g). Consistent with the effects of crude venom in the chick biventer cervicis nerve-muscle assay, toxin-induced responses in neuroblastoma cells were abolished by TTX (Control response, 3.12 ± 0.04 AFU; TTX (1 μM), 0.12 ± 0.03 AFU), suggesting direct effects on voltage-gated sodium channels (Figure 1e). The amino acid sequence of the active component, which we called calliotoxin or δ-elapitoxin-Cb1a, was determined as: LE**C**YDTIFKWHTMT**C**PEGQNL**C**FYYFTWRIFLVRG**C**TAT**C**PVGYSHTH**CC**DTDK**C**NN using a combination of Edman degradation sequencing and venom gland transcriptome analysis (Figure 2).

The sequence calculated monotopic molecular weight is 6725.91 Da, which is in accord with the mass spectrometry monoisotopic molecular weight of 6725.9 Da. Calliotoxin belongs to the class of three-finger toxins (3FTx) but has low sequence homology to other known toxins in the family (Figure 2).

**Figure 3:**
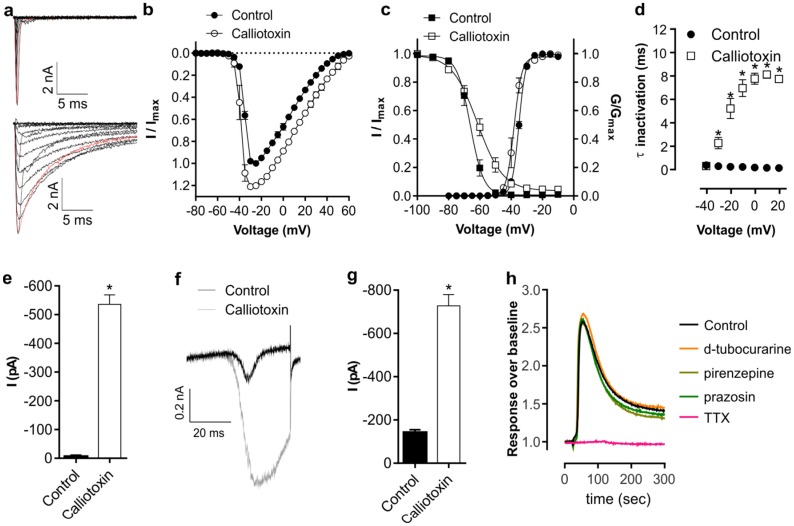
Activity of calliotoxin (200 nM) in HEK-293 cells heterologously expressing hNa_V_1.4 assessed by automated patch clamping. (**a**) Representative trace of sodium currents before (upper) and after addition of calliotoxin (lower) elicited by depolarizing steps between −80 and +60 mV in 10 mV increments. The red trace highlights the depolarizing step to −20 mV; (**b**) Current-voltage relationship before and after the addition of calliotoxin; (**c**) Voltage-dependence of activation (circles) and voltage-dependence of steady-state fast inactivation (squares) before and after addition of calliotoxin; (**d**) Voltage-dependence of fast inactivation time constants before and after addition of calliotoxin; (**e**) Average persistent current elicited 40–50 ms after a −20 mV depolarizing step before and after addition of calliotoxin; (**f**) Representative trace of ramp sodium current before and after addition of calliotoxin; (**g**) Peak inward current elicited by a depolarizing ramp (2.4 mV/ms) before and after addition of calliotoxin. Data are shown as mean ± SEM with *n* = 3 replicates; (**h**) Calliotoxin-induced effects are mediated through Na_V_ channels. Calliotoxin-induced Ca^2+^ responses in SH-SY5Y cells were inhibited by tetrodotoxin (TTX) (1 μM) but not the nAChR antagonist d-tubocurarine (10 μM), the M1 mAChR antagonist pirenzepine (100 μM), or the α1-adrenoreceptor antagonist prazosin (10 μM).

3FTxs have diversified into forms with novel pharmacology that include post-synaptic (muscarinic and nicotinic receptors), synaptic (acetylcholinesterase inhibitors), and presynaptic (L-type calcium channels) activities [61,82]. However, none to date have been reported with activity at voltage-gated sodium channels. Therefore, we further examined the pharmacological effect of calliotoxin on HEK293 cells heterologously expressing Na_V_1.4, the Na_V_ subtype essential for skeletal muscle function in mammals, using whole-cell patch-clamp recordings. Calliotoxin enhanced peak inward current and delayed inactivation (Figure 3a,b), causing a small but significant hyperpolarizing shift in the V_1/2_ of activation (control, −35.0 ± 0.1 mV; calliotoxin, −38.1 ± 0.3 mV; *p* < 0.0001) and a significant depolarizing shift in the V_1/2_ of fast inactivation (control, −66.1 ± 0.5 mV; calliotoxin, −61.6 ± 0.7 mV; *p* < 0.0001) (Figure 3c). Calliotoxin voltage-dependently delayed the inactivation time constant (−20 mV pulse: control, 0.24 ± 0.02 ms; calliotoxin, 5.22 ± 0.9 ms; *p* < 0.05; Figure 3d), which resulted in a persistent current (−20 mV pulse: control, −6 ± 4 pA; calliotoxin, −536 ± 33 pA; *p* = 0.015; Figure 3e). In line with the above Na_V_ activator activity, calliotoxin also significantly increased inward ramp currents (control, −143 ± 12 pA; calliotoxin, −728 ± 52 pA; *p* = 0.0004; Figure 3f,g).

These results demonstrate that calliotoxin acts directly at Na_V_ in a manner reminiscent of other Na_V_ activators from the venom of cone snails, scorpions, spiders, wasps, and anemones [26,83,84,85,86,87,88,89,90]. The potency is similar to that characterised for Australian hexathelid spiders such as funnel webs (*Atrax* and *Hadronyche* species) and the eastern mouse spider, which produce similar fasciculations in the neuromuscular organ bath assay (*Missulena bradleyi*) [91,92] (Figure 1c). Thus, calliotoxin is the first identified Na_V_ activator from snake venom and represents a structurally novel class of Na_V_ gating modifiers. While crotamine peptides from South American *Crotalus* species (rattlesnakes) had been previously thought to interact with sodium channels [93,94,95], in addition to being potent myotoxins, it has been recently observed that the neurotoxic activity is guided by potassium channel interactions [96,97,98,99]. A PLA_2_ toxin previously isolated from the Asian pit-viper *Gloydius ussurensis* showed voltage-dependence of activation of sodium channels in sensory neurons [100]. However, in contrast to calliotoxin, this toxin caused predominant effects on the voltage-dependence of activation and decreased peak current. In addition, this toxin also affected voltage-gated potassium channels. It remains to be determined whether calliotoxin also has effects on other members of the voltage-gated ion channel family.

There is a general evolutionary trend for an inverse relationship between mechanical forms of prey subjugation and chemical forms. In some predatory lineages, once a mechanical form of prey capture has evolved, it is co-opted for use as a delivery system for venom. Spiders are one such example, in which the ancestral mechanical form of predation using chelicerae to inflict fatal wounds in prey items resulted in a selection pressure for the derived use as a venom delivery system, with a subsequent reduction in chelicerae size [101,102]. Similarly, the elongated dentition used by cleaner wrasse mimics from the *Plagiotremus* fangblenny genus for their unique parasitic feeding strategy was co-opted for venom delivery in the *Meiacanthus* genus of fangblenny [103,104]. In other cases, a structural feature has been derived to become a venom-delivering apparatus, such as the fin-supporting spines in fish [105]. In early toxicoferan venomous reptiles, the pre-existing teeth were sufficient for the generation of a wound, allowing for low-pressure venom delivery via chewing, with the array of extant dentition types subsequently evolving as refinements [27,58]. An alternate scenario is one in which a hypertrophic mechanical structure is atrophied subsequent to the evolution of a different part of the anatomy to serve as a venom-delivering structure. Examples include the large claws of plesiomorphic scorpions accompanied by small stingers (telsons), relative to the gracile claws of apomorphic scorpions which have large telsons [106], and octopus beaks and glands, with species having large beaks having smaller glands than those which have small beaks [107,108].

The primary function of predatory venoms is the subjugation of prey items rather than lethality [27]. From an evolutionary and practical perspective, there is little difference between an unconscious or completely immobilized prey item and a dead one as the helpless former would simply suffocate in the stomach after being swallowed. Thus, the primary shaping pressure in predatory venom evolution is rapid prey subjugation rather than rapid lethality [1].

Chemical forms of prey subjugation allow for a decoupling of the physical interaction between predator and prey. Snakes that hunt animals capable of inflicting serious retaliatory wounds often release their intended prey after envenomation. In this situation, selection may favour the evolution of toxins that rapidly disable prey, either to prevent it from moving too far to be recovered or to prevent the possibility of it attacking and injuring the snake. For example, Northern Pacific rattlesnakes (*Crotalus oreganus*) and inland taipans (*Oxyuranus microlepidotus*) both feed on rodents, which are capable of inflicting life-threatening bite wounds upon snakes. Both species release their prey after biting. Crotamine, a neurotoxin in the venom of the rattlesnake, paralyses the hind limbs of the rodent in seconds, ensuring it is unable to travel far from the heavy bodied ambush-hunting snake in the approximately two minutes it takes for it to die [109,110]. Inland taipans, which are fast moving active hunters, face their rodent prey in confined underground spaces and thus are at considerable risk of sustaining retaliatory injuries. Their venom is the most toxic (to rodents) of any snake [111] and contains an exceptionally fast acting fXa:fVa coagulation enzyme complex, which causes small clots to form throughout the rat’s circulatory system resulting in rapid knockdown via stroke injury [27,112,113,114]. In human bite victims the same amount of venom is diluted into a much larger blood volume, producing countless microthrombi which by themselves are too small to cause a stroke but instead consume all of the clotting factors, with death the result of internal bleeding such as cerebral hemorrhage [115].

Rapid knockdown effects may also evolve in situations where the cost of prey recovery is particularly high due to the distance that might be traversed by dying prey. Mambas (genus *Dendroaspis*) are arboreal elapid snakes that feed on birds, which they subdue with venom containing unique kunitz peptide neurotoxins that rapidly cause excitatory paralysis through the inhibition of voltage-dependent potassium channels, resulting in the sustained release of acetylcholine, while 3FTx inhibit acetylcholinesterase, further elongating the acetylcholine action [27,82,116,117,118,119,120]. Another species of arboreal elapid snake, the Stephens’ banded snake (*Hoplocephalus stephensi*), uses natriuretic peptide toxins to cause a precipitous drop in the blood pressure of prey animals [121]. In the case of cone snails, the venom was first evolved for worm-hunting, with the subsequent evolution of fish-specific venom for defence against predators, which was then co-opted on at least two occasions for fish-specialist prey preference [122,123,124]. Piscivorus cone snails also risk being unable to recover their prey if the fish are able to escape after envenomation—even if the fish only swims a small distance before dying, the slow-moving snail faces a high risk of losing its meal to an opportune scavenger. The risk of going hungry thus forms the selection pressure driving the evolution of the devastating chemical weaponry of venoms.

Na_V_ are widely expressed across animal phyla including in the invertebrate and vertebrate nervous systems and are critically important for neuromuscular action potential conduction. Accordingly, toxins that target Na_V_ are widely found in venoms, with known families including the µ-conotoxins, µ-theraphotoxins, δ-conotoxins, α- and β- scorpion toxins, and δ-theraphotoxins [26,83,84,85,86,87,88,89,90]. Although the mechanism of action of these toxins is distinct, all impair normal Na_V_ function, which critically depends on voltage-dependent activation and inactivation to allow regulated Na^+^ influx. As a consequence, these toxins lead to catastrophic disruption of neuronal or neuromuscular physiology. Although it initially seems surprising that a snake toxin would converge on the same activity as that of toxins from cone snails, spiders, wasps, and scorpions, convergence in molecular targeting is actually common amongst venomous organisms [26].

On a deeper theoretical level, these results have relevance to the debate regarding whether evolution is contingent or predictable (e.g., [125,126]). In fact, as Daniel Dennett has pointed out [127], it is both. Targeting Na_V_ is, in Dennett’s terminology, a “Good Trick”. Since there is likely to be a limited number of good tricks available to venomous organisms with the need to rapidly render prey incapable of escape or retaliation, it is predictable that convergence will occur [1]. The possibilities for evolutionary innovation, however, are constrained by historical contingency [128]. It is historical contingency that makes it more likely (thus more predictable) that the novel activity observed in the present study should emerge within the 3FTx, a toxin class typically under strong positive selection within the Elapidae [129]. Thus we see a predictable convergence of molecular targeting arising (predictably) within a toxin class “selected” by historical contingency—evolution is not predictable rather than contingent, it is predictable *because* it is contingent.

In this case, the selection pressure driving this contingent and predictable evolutionary trajectory is involvement with a prey animal that relies upon extreme coordination in its fight or flight response. Cone snails have such an interaction with fish, mamba snakes with birds, and long-glanded blue coral snakes with fast moving snakes, some of which are capable of venomous retaliatory actions potentially lethal to the coral snakes. Thus, a novel, extreme selection pressure has driven the venom evolution in a new direction. The hallmark of other elapid snake venoms are α-neurotoxins that antagonistically bind to the post-synaptic nicotinic acetylcholine receptors to produce flaccid paralysis, as is also the case in other venomous snakes lineages including gracile colubrid snakes with enlarged rear-fangs [19,27,44,47,59,62,130,131,132,133,134,135,136]. Potent α-neurotoxicity preceded the evolution of the high-pressure front-fanged system of elapids, as was revealed by our discovery of α-colubritoxin, the first 3FTx isolated and characterized from a non-front-fanged lineage [59]. The toxin was of the same form as had been studied in elapid snakes previously, where they had been called ‘weak neurotoxins’ since they were only weakly potent on mice [137,138,139,140,141,142,143,144,145,146,147,148,149]. A problem that had also confounded studies of *Boiga irregularis* (brown tree snake), which had thus concluded they were non-venomous based on their effects in a murine model [150,151,152,153,154,155]. However, all such plesiotypic 3FTx rich venoms were later shown to be much more potent in a taxon-specific manner on diapsid (bird/reptile) than on synapsid (mammal) post-synaptic nicotinic acetylcholine receptors, with some non-front-fanged snakes being as potent as elapid snakes in such a taxon specific manner and thus were not ‘weak’ against natural prey items [59,82,130,156,157,158,159,160].

In contrast, *C. bivirgatus* venom produces a new action with a net effect that is diametrically opposed to the typical elapid snake mode of action: spastic paralysis that is potent on both the avian and murine assays models used in this study. The early evolving elapid snake neurotoxic effect is one of respiratory failure from a paralysed diaphragm because the snake toxins prevent acetylcholine facilitating the diaphragm’s contraction, thus leaving it stuck in the non-contracted resting state. In contrast, *C. bivirgatus* venom keeps sodium channel toxins open, blocking the closing of the channel that would terminate the nerve transmission and allow the muscle to go back to the resting state. In this case, the net effect is that muscles are stuck in the contracted activated state instead of the typical elapid effect of being stuck in the non-contracted resting state. This is convergent with the rigid paralysis cone snails produce when feeding upon fish or the muscle fasciculations characteristic of mamba envenomations, both of which have been shaped by prey with high escape potential. The unique selection pressure operating with the niche occupied by *C. bivirgatus* has resulted in the flipping of the neurotoxic polarity, with this novel effect evolving due to the selection pressure of fast moving prey with high escape potential but also the potential for lethal retaliatory actions.

While the speed of action, and thus prey subjugation, are rapid, the question of relative toxicity must still be considered. Why else would sodium channel delay of inactivation toxins not be more common and only selected for in such extreme circumstances? Why has this only emerged once in snakes? It very well may be that while the α-neurotoxins are slower to take effect, they may be ultimately more effective as a consequence of their ability for sustained effect due to the pathology being produced due to simple steric inhibition. This is reflective in *C. bivirgatus* having a murine intravenous LD_50_ of 0.7–0.8 mg/kg [81] in comparison to that of a venom almost exclusively containing classic α-neurotoxins such as *Acanthophis antarcticus* which has a murine intravenous LD_50_ of 0.25 mg/kg [159,160]. Consistent with this, the black mamba (*Dendroaspis polylepis*), which consume a higher percentage of rodents than birds in their diet, has a murine intravenous LD_50_ of 0.5 mg/kg, while the green mambas, which have a higher percentage of birds in their diets, and thus would benefit from less toxic but more rapidly acting knock-down venoms, have LD_50_s of over 2.5 mg/kg for the Eastern green mamba (*Dendroaspis angusticeps*), 0.9 mg/kg for the Jameson’s mamba (*Dendroaspis jamesoni*), and 1 mg/kg for the Western green mamba (*Dendroaspis viridis*) [161]. Such tests reinforce that α-neurotoxicity is likely to be selected for due to its sustained paralytic effect, which is ultimately lethal due to its persistence; whereby complete immobilization also results in death but with the former being the outcome selected for by the shaping evolutionary pressures as there is no functional difference between a prey in suspended animation and one that is deceased.

It is interesting to note that unlike plesiotypic 3FTx which retain all 10 ancestral cysteines and are more potent to avian/reptilian post-synaptic nicotinic acetylcholine receptors than those of mammals [59,61,82,130,132,134,135], the *C. bivirgatus* plesiotypic presynaptic sodium channel toxins do not display taxon specific effects, as the crude venom and pure toxins were potent on both avian neuromuscular organ bath preparations and mammalian ion-channels FLIPR assays (Figure 1 and Figure 3) . Calliotoxin lacks the second and third plesiotypic cysteines (Figure 2), similar to that of the classic Type I (aka: short-chain) α-neurotoxins, which also do not display a taxon specific effect [61,160]. This is consistent with the fact that while there are very few human *C. bivirgatus* envenomations on record, the majority were lethal. Thus, while the venom may be three times less potent than that of α-neurotoxic snakes, this is off-set by the massive venom yields produce by the elongated venom glands, with dry weight single venom extraction quantities reaching 150 mg in this study for large (>1.2 m) specimens. This massive venom yield proportional to the length of its slender body is produced by the innovatively slender venom glands connected to the fang by a venom duct that extends until the venom gland is inside the rib cavity (Figure 1b) with the venom gland compressing musculature now wrapped completely around the gland, squeezing it from the back third in a manner analogous to that of the cone snail venom system. Thus the venom diversification in this lineage is paralleled by a morphological uniqueness, the evolution of which may have been driven by the same selection pressures as that of the toxins themselves. Studies between this species and the short-glanded *C. gracilis* and the long-glanded *C. intestinalis* will be revealing in regards to timing the venom gland elongation versus the timing of the molecular evolution of this extremely novel new diversification of the 3FTx framework. Regardless of the relative timing, the rapid interplay underscores that reptiles as a general character are extremely morphologically plastic and that their venom systems also dynamically evolve at all trophic levels.

A crucial difference between the two paralytic strategies (inhibitory/flaccid/limp or excitatory/contracting/spastic) rests upon how long the effect can be sustained. In the case of nerve transmission, certain ions move along an ion-gradient whenever a particular channel is opened at any number of steps in the transmission of a nerve impulse. Over-stimulation has inbuilt rate-limiting steps as the ion-pumps can only work at a finite rate to reset the ion-gradient. Thus, keeping the sodium channel open by delaying its inactivation, the long-glanded blue coral snake toxins will sustain this action only so long as there is a sufficient sodium gradient. Once isotonic levels for this ion are reached, there will be no more spontaneous action until the ion pumps re-establish a sufficient gradient. Thus, the delay of presynaptic sodium channel inactivation is a resource which may be exhausted. In contrast, α-neurotoxins bind to and block the post-synaptic nicotinic acetylcholine receptors; an action that may be sustained for a much longer period as it does not require the involvement of other molecules or chemical gradients. It is a case of antagonistic binding to the receptors, with fundamental interactions such as electrostatic charge or hydrophobic attractions determining the interaction strength. Once bound to the receptor, some toxins are virtually irreversible. Such immobilizing paralysis would subjugate the prey items quite efficiently for an indefinite period, thus having a marked degree of lethality. Therefore, there would exist a strong selection pressure to maintain such a predatory weapon. This underscores just how radical a change in functionality has occurred in *C. bivirgatus* venom.

The shift also parallels a shift in the metabolic state of the prey. Many Australian elapids snakes feed on reptilian prey that is inactive/dormant at the time of the snake’s attack, and thus suffocation through lethal lethargy inducing flaccid paralysis is an effective predatory strategy in elapid snakes [56]. Such a predatory strategy is also widely used in a myriad of venomous snakes that lack the apomorphic high-pressure delivery systems [44,47,59,62,130,131,132,134,135,136]. In contrast, the long-glanded blue coral snake feeds on other snakes that are at their highest activity level. Thus, the rapidity of effect becomes the primary selection pressure [1].

Due to its extraordinary venom glands and extremely novel venom chemistry, this enigmatic species may be considered as the epitome of toxicoferan reptile venom system derivation. Study of this species increases our knowledge of venom evolution and demonstrates the relevance of studying toxins, proteins unconstrained by endophysiological functions and under extreme selection pressures [56], in order to gain insight into more general patterns of evolution. In addition, the results are of considerable interest to those seeking to understand the mechanisms underlying Na_V_ gating. These results underscore the dynamic evolution of the Toxicofera reptile system at all trophic levels and reinforces the value of using evolution as a roadmap for biodiscovery.

## 3. Materials and Methods

### 3.1. Materials

Specimens of the long-glanded blue coral snake (*Calliophis bivirgatus*) were captive animals of Malaysia stock. All other reagents were from Sigma Aldrich (Castle Hill, Sydney, NSW, Australia) unless otherwise specified.

### 3.2. Animal Ethics

All animal experiments used in this study were approved by the SOBS-B Monash University Animal Ethics Committee MARP/2014/97 (1 December 2014).

### 3.3. Neurotoxicity Studies

Male chicks (4–10 days) were euthanised by CO_2_ and exsanguination. Both chick biventer cervicis nerve muscle preparations were isolated and mounted on wire tissue holders under 1 g resting tension in 5 mL organ baths containing physiological salt solution (NaCl, 118.4 mM; KCl, 4.7 mM; MgSO_4_, 1.2 mM; KH2PO_4_, 1.2 mM; CaCl_2_, 2.5 mM; NaHCO_3_, and 25 mM glucose, 11.1 mM), maintained at 34 °C and bubbled with 95% O_2_/5% CO_2_. Indirect twitches were evoked by electrical stimulation of the motor nerve (supramaximal voltage, 0.2 ms, 0.1 Hz) using a Grass S88 stimulator (Grass Instruments, Quincy, MA, USA). d-Tubocurarine (10 μM) was added, and subsequent abolition of twitches confirmed selective stimulation of the motor nerve, after which thorough washing with physiological salt solution was applied to re-establish twitches. The preparation was equilibrated for 30 min before the addition of venom, which was left in contact with the preparation for a maximum of 3 h to test for slow developing effects. Efficacy of tetrodotoxin (TTX; 0.1 μM) was assessed via a 10 min pre-incubation in the organ bath.

#### 3.3.1. Assay-Guided Fractionation

Crude *Calliophis bivirgatus* venom (150 µg) was fractionated into thirty second fractions on a ThermoScientific Hypersil (Scoresby, Vic, Australia) BDS C18 column (200 mm, 5 µm) using a linear gradient from 5% to 70% solvent B over 60 min (solvent A, H_2_O/0.05% TFA; solvent B, 90% acetonitrile/0.05% TFA) with a flow rate of 1 mL/min. Orthogonal purification of active fractions was carried out using a Phenomenex Synergi Hydro-RP 80A HPLC column (Phenomenex, Torrance, CA, USA) (250 × 4.6 mm, 4 µm) with flow rate of 0.7 mL/min and linear gradient from 30% to 50% B over 40 min (solvent A, H_2_O/0.05% TFA; solvent B, 90% acetonitrile/0.05% TFA). Activity testing was conducted as previously described [162] in human neuroblastoma SH-SY5Y cells (ECACC, Salisbury, Wiltshire, UK) maintained at 37 °C/5% CO_2_ in Roswell Park Memorial Institute (RPMI) medium containing 15% foetal bovine serum (FBS) and 2 mM l-glutamine. Cells were loaded with Calcium 4 No-Wash dye (Molecular Devices, Sunnvale, CA, USA) in 384-well black-walled imaging plates (Sigma Aldrich, Castle Hill, NSW, Australia) 48 h after plating at a density of 50,000 cells/well and incubated for 30 min at 37 °C. Freeze-dried fractions were resuspended in physiological salt solution (PSS; composition: 140 mM NaCl, 11.5 mM glucose, 5.9 mM KCl, 1.4 mM MgCl_2_, 1.2 mM NaH_2_PO_4_, 5 mM NaHCO_3_, 1.8 mM CaCl_2_, 10 mM 4-(2-hydroxyethyl)-1-piperazineethanesulfonic acid (HEPES), pH 7.4) and added to dye-loaded cells using a FLIPR^TETRA^ (Molecular Devices, Sunnyvale, CA, USA) plate reader while measuring real-time fluorescence responses (excitation, 470–495 nm; emission, 515–575 nm). Selective activation of endogenously expressed Na_V_ channels was confirmed through inhibition of calliotoxin-induced responses by TTX (1 μM) and lack of effect of the nAChR antagonist d-tubocurarine (10 μM), the M1 mAChR antagonist pirenzepine (100 μM) and the α1-adrenoreceptor antagonist prazosin (10 μM) (Figure 3h).

#### 3.3.2. Mass Spectrometry

Samples were separated using reversed-phase chromatography on a Dionex Ultimate 3000 RSLC nano-system. Using a flow rate of 30 µL/min, samples were desalted on a Thermo PepMap 100 C18 trap (0.3 × 5 mm, 5 µm) for 5 min, followed by separation on a Acclaim PepMap RSLC C18 (150 mm × 75 µm) column at a flow rate of 300 nL/min with a gradient of 10%–95% buffer B over 60 min where buffer A = 1% ACN/0.1% FA and buffer B = 80% ACN/0.1% FA. Eluted molecules were directly analysed on an Orbitap Elite mass spectrometer (Thermo, Brisbane, Australia) using an NSI electrospray interface. Source parameters included a capillary temperature of 275 °C; S-Lens RF level at 60%; source voltage of 2 kV and maximum injection times of 200 ms for MS. Data were deconvoluted using Protein Deconvolution software (Thermo).

#### 3.3.3. Edman Degradation and Venom Gland Transcriptomics

Edman degradation was carried out by the Australian Proteome Analysis Facility (APAF, Sydney, Australia). Purified native calliotoxin was solubilised in ammonium bicarbonate (25 mM)/10% ACN and reduced using DTT (25 mM) at 56 °C for 0.5 h, followed by alkylation using iodoacetamide (55 mM) at room temperature for 0.5 h. The reaction mix was then desalted/purified by RP-HPLC using a Zorbax 300SB-C18 column (3 × 150 mm, Agilent, Santa Clara, CA, USA). The volume was reduced under vacuum and loaded onto a precycled, Biobrene-treated disc and subjected to 60 cycles of Edman *N*-terminal sequencing using an Applied Biosystems 494 Procise Protein Sequencing System (Applied Biosystems, Foster City, CA, USA), resulting in unambiguous identification of 47 amino acid residues. Venom gland transcriptomics were conducted by the IMB Sequencing Facility (Institute for Molecular Bioscience, The University of Queensland, St Lucia, Qld, Australia). Libraries were prepared with the TruSeq Stranded mRNA kit (Illumina, San Diego, CA, USA), and were sequenced on the Illumina NextSeq (Illumina) 500 using 2 × 150 bp reads and V2 chemistry. To identify the full sequence of δ-elapitoxin-Cb1a, forward and reverse sequences were merged using MacQIIME (Werner Lab, SUNY Cortland, NY, USA) join_paired_end.py and matched to the sequence determined by Edman degradation using standalone BLAST.

#### 3.3.4. Electrophysiology

HEK-293 cells stably expressing hNa_V_1.4 (SB Drug Discovery, Glasgow, UK) were cultured in MEM containing 10% *v*/*v* foetal bovine serum, supplemented with l-glutamine 2 mM and selection antibiotics, as recommended by the manufacturer. Cells were grown in a humidified 5% CO_2_ incubator at 37 °C, grown to 70%–80% confluence, and passaged every three to four days using TrypLE Express (Invitrogen, Scoresby, VIC, Australia). For electrophysiology experiments, cells were dissociated by incubating with Detachin (Bio-Scientific, Kirrawee, NSW, Australia) at 37 °C for 5 min, then resuspended in Ex-Cell ACF CHO Medium with 25 mM HEPES (Sigma-Aldrich, Castle Hill, NSW, Australia) and allowed to recover with stirring for 30 min.

Whole-cell patch-clamp experiments were performed on a QPatch-16 automated electrophysiology platform (Sophion Bioscience, Ballerup, Denmark) using 16-channel planar patch chip plates (QPlates; Sophion Bioscience, Ballerup, Denmark) with a patch hole diameter of 1 µm and resistance of 2 ± 0.03 MΩ. Cell positioning and sealing parameters were set as follows: positioning pressure −60 mbar, minimum seal resistance 0.1 GΩ, holding potential −100 mV, holding pressure −20 mbar. Whole-cell currents were filtered at 5 kHz and acquired at 25 kHz.

The extracellular solution contained in mM: NaCl 145, KCl 4, CaCl_2_ 2, MgCl_2_ 1, HEPES 10, and glucose 10; pH 7.4; osmolarity 305 mOsm. The intracellular solution contained in mM: CsF 140, EGTA/CsOH 1/5, HEPES 10 and NaCl 10; pH 7.3; osmolarity 320 mOsm. Purified native calliotoxin was diluted in extracellular solution to a concentration of 200 nM and incubated for 5 min. The effects of calliotoxin were compared to pre-toxin control parameters in the same cell.

Current (I)-voltage (V) curves were obtained with a holding potential of −80 mV followed by a pre-pulse of −100 mV for 50 ms and a series of 50 ms step pulses that ranged from −80 to +60 mV in 5 mV increments before returning to a holding potential of −80 mV (repetition interval 5 s). Conductance-voltage curves were obtained by calculating the conductance (G) at each voltage (V) using the equation *G* = *I*/(*V* − *V_rev_*), where *V_rev_* is the reversal potential, and fitted with a Boltzmann equation: G_Na_ = G_Na,max_/1 + exp[(V_m_ − V_1/2_)/*k*], where G_Na_ is the voltage-dependent sodium conductance, G_Na,max_ is the maximal sodium conductance, V_1/2_ is the potential at which activation is half-maximal, V_m_ is the membrane potential, and *k* is the slope factor. Fast inactivation time constants were calculated by fitting current decay traces obtained from the above I-V protocol with a single exponential function, and persistent current was determined as the average current 40–50 ms after pulse onset.

Voltage dependence of steady-state fast inactivation was measured using a series of 500 ms pre-pulses, ranging from −120 to −10 mV in 10 mV increments, followed by a 20 ms pulse of −20 mV to assess the available non-inactivated channels (repetition interval 30 s). Peak inward currents (I) were normalized to the maximal inward current (I_max_) and fitted using a Boltzmann equation: I/I_max_ = 1/(1 + exp[(V_m_ − V_1/2_)/*k*)], where I_max_ is the maximal inward current, V_1/2_ is the half-maximal sodium current, V_m_ is the pre-conditioning pulse potential, and *k* is the slope factor.

Ramp currents were evoked by a depolarization from a holding potential of −100 to +20 mV at a rate of 2.4 mV/ms.

### 3.4. Data Analysis and Statistics

Unless otherwise stated, all data are expressed as the mean ± standard error of the mean (SEM) determined from at least *n* = 3 replicates. FLIPR^Tetra^ results were converted to response over baseline using ScreenWorks 3.2.0.14 (Molecular Devices, Sunnyvale, CA, USA) and plotted using GraphPad Prism 6 (GraphPad Software, San Diego, CA, USA). Twitch heights were measured from the baseline in two minute intervals. Responses were expressed as a percentage of twitch height prior to the addition of the peptide. Contractile responses to agonists obtained at the conclusion of the experiment were measured and expressed as a percentage of the response obtained prior to the addition of peptide. Where indicated, a one-way analysis of variance (ANOVA) followed by a Bonferroni-corrected post-hoc test was used to determine statistical significance of responses. Statistical analysis was performed using the GraphPad Prism 5.
